# Beyond the boundary: a new road to improve photosynthesis via wind

**DOI:** 10.1093/jxb/eraf325

**Published:** 2025-07-15

**Authors:** Killian Dupont, Tomas E van Den Berg, Jiayu Zhang, Arnold F Moene, Silvère R M Vialet-Chabrand

**Affiliations:** Horticulture and Product Physiology (HPP), Wageningen University, P.O. Box 16, Wageningen 6700 AA, The Netherlands; Horticulture and Product Physiology (HPP), Wageningen University, P.O. Box 16, Wageningen 6700 AA, The Netherlands; Horticulture and Product Physiology (HPP), Wageningen University, P.O. Box 16, Wageningen 6700 AA, The Netherlands; Meteorology & Air Quality Group (MAQ), Wageningen University, P.O. Box 47, Wageningen 6700 AA, The Netherlands; Horticulture and Product Physiology (HPP), Wageningen University, P.O. Box 16, Wageningen 6700 AA, The Netherlands; University of Nottingham, UK

**Keywords:** Airflow, controlled-environment agriculture (CEA), gas exchange, heat exchange, leaf boundary layer, leaf size, microclimatic gradient, photosynthesis, stomatal conductance, transpiration, wind

## Abstract

Plants interact with their microclimate, simultaneously responding to and influencing it. A key element in this interaction is the leaf boundary layer: a stagnant air layer enveloping the leaf, creating a resistance to heat and gas exchange. Its thickness, altered mainly by airflow and leaf morphology, determines the leaf-to-air interaction. Field crops experience wind speeds of 0–8 m s^−1^ at the canopy top, with wind gusts up to 20 m s^−1^, but wind speeds drop significantly within the canopy, creating localized low-airflow conditions. Conversely, indoor-grown crops always encounter low wind speeds (0–1 m s^−1^) and these, especially with larger leaves, restrict heat and gas exchange, impacting photosynthesis and transpiration. Although the effect of the leaf boundary layer on plant exchange processes has been defined, its magnitude remains poorly characterized and is frequently underestimated. This review re-examines its role, and underlying processes are further explained by using an existing modelling approach informed by published physiological parameters from relevant crops. This model suggests that in greenhouses, increases in wind speed typically smaller than 0.2 m s^−1^ could boost diurnal photosynthesis by 10–20%, although with possible detrimental side-effects such as growth reductions due to mechanical effects and excessive transpiration. In the field, leaves within the canopy often experience thick boundary layers (conductance <0.5 mol m^−2^ s^−1^). The role of the boundary layer needs to be re-evaluated, but this will require new tools, methods, and models to make a breakthrough in understanding this overlooked process in both field and controlled-environment agriculture crops.

## Teaser

Environmental factors such as wind and plant-specific traits including leaf size can be manipulated to alleviate limitations imposed on photosynthesis by low leaf boundary layer conductance.

## Introduction

Meeting future food demands requires improving crop yields, with photosynthesis being a central target for improvement through enhanced chloroplast biochemistry and CO_2_ diffusion from the atmosphere to the chloroplast (Zhu *et al*., 2010; Long *et al*., 2015; Bailey-Serres *et al*., 2019; Wu *et al*., 2019; Croce *et al*., 2024). This ‘CO_2_ supply chain’ is an indirect breeding target (Fischer and Rebetzke, 2018) and is regulated by the conductances of the boundary layer ($g_{b}$), stomata ($g_{s}$), and mesophyll ($g_{m}$) acting in series, which controls gas diffusion along the pathway (Flexas *et al*., 2012; Franks *et al*., 2015; Cano *et al*., 2019; Deans *et al*., 2019; Salesse-Smith *et al*., 2024).

Genetic strategies advance our ability to improve these conductances through traditional breeding, natural genetic variation, molecular tools, and phenotyping approaches (Rebetzke *et al*., 2012; Franks *et al*., 2015; Faralli *et al*., 2019; Pan *et al*., 2022; Al-Salman *et al*., 2023; Salesse-Smith *et al*., 2024). However, plant phenotype and performance are also highly influenced by environmental conditions (De Gelder *et al*., 2012; Reynolds and Langridge, 2016; Köhler *et al*., 2019; Burroughs *et al*., 2023). To achieve future yield improvements, it is essential to understand how environmental factors influence photosynthesis (Murchie *et al*., 2018) at each stage of the CO_2_ supply chain.

Fluctuating environmental conditions in the field directly impact CO_2_ transport and photosynthesis, influencing overall plant performance. In contrast, controlled environments such as greenhouses and vertical farms offer precise regulation of conditions such as light, temperature, and CO_2_ (SharathKumar *et al*., 2020; Poorter *et al*., 2023). These artificial settings differ markedly from the field, complicating direct comparisons (Poorter *et al*., 2016). To bridge this gap, recent research has focused on mimicking natural fluctuations, primarily in light intensity (Kaiser *et al*., 2015; Vialet-Chabrand *et al*., 2017), with growing interest in using dynamic environmental control to improve resource-use efficiency in vertical farming systems (Kaiser *et al*., 2024; Bechtold *et al*., 2025). However, fluctuations of other factors such as CO_2_ concentration, temperature, humidity, and especially airflow remain comparatively understudied (Leakey *et al*., 2003; Yamori, 2016; Ainsworth and Long, 2021).

Airflow in controlled environment agriculture (CEA) is rarely reported and often not actively controlled, although studies have highlighted its importance in the field (Cleugh, 1998; Gardiner *et al*., 2016). The lack of airflow data could represent a missed opportunity to understand how it influences photosynthesis. The leaf boundary layer, a thin layer of stagnant air that forms around leaves, plays a crucial role in determining heat and gas exchange between the leaf surface and the surrounding canopy environment, and is strongly influenced by airflow. The boundary layer creates a physical barrier for mass and heat transfer from and to the leaf surface (Schuepp, 1993). While its physical effects are well understood, its precise impact on limiting photosynthesis, especially in low-airflow environments, is poorly quantified.

Only a few studies have acknowledged the potential photosynthesis limitation due to limited mass transfer (Jarvis and McNaughton, 1986; Bunce, 1988; Grace, 1988; Collatz *et al*., 1991). Experimental evidence demonstrates that short-term (minutes to hours) increases in wind can increase steady-state photosynthesis by 5–30% due to substantial removal of the boundary layer (Bunce, 1988; Aphalo and Jarvis, 1993; Schymanski and Or, 2016). Modelling studies have indicated that $g_{b}$ modulates leaf temperature, transpiration, and photosynthesis rate, especially during midday depressions where $g_{b}$ can impact photosynthesis by 25–65% (Collatz *et al*., 1991; Aphalo and Jarvis, 1993). More recent studies have further stressed the role of the boundary layer in the uniformity of greenhouse microclimates and the accuracy of remote sensing, due to its substantial impact on the plant’s energy balance (Leigh *et al*., 2017; Kimura *et al*., 2020a, 2024), and in selecting traits such as leaf width that influence water-use efficiency (WUE) (Cano *et al*., 2019; Pan *et al*., 2022; Al-Salman *et al*., 2023; Tamang *et al*., 2023). Given its often overlooked but significant role in regulating heat and gas exchange, the boundary layer deserves renewed focus to fully understand its impact on photosynthesis.

This review addresses the question, ‘how much does the leaf boundary layer limit photosynthesis, and how can this be quantified?’. The specific target is low-wind speed environments (0–2 m s^−1^), which have the largest impact on $g_{b}$. Low wind is typical in CEA and consequently greater emphasis is placed on these crops, but it can also occur in field crops, especially within the canopy. We first introduce the relationships between the leaf boundary layer and heat and gas exchange, and their connection to airflow. A critical examination is then made of existing formulas that predict the leaf boundary layer and we consider their limitations. An overview is provided of leaf boundary layer conductance values for a variety of CEA and field crops. Finally, we quantify limitations on leaf-level photosynthesis and transpiration by means of an existing approach using a combined photosynthesis, stomatal conductance, and energy balance model for both greenhouse and field scenarios.

## Environmental and plant-controlled exchange processes

Jarvis and McNaughton (1986) highlighted a longstanding distinction between the perspectives of micrometeorologists and plant physiologists regarding plant–atmosphere interactions. Micrometeorologists typically treat the canopy as a single entity, focusing on its influence on the atmosphere and modelling water fluxes using canopy conductance, an aggregate representation of stomatal responses modulated by transfer processes and weather variables such as radiation and humidity (Ronda *et al*., 2001; McVicar *et al*., 2012; Boussetta *et al*., 2013; Zeng *et al*., 2019). In contrast, plant physiologists often emphasize biological control at the leaf level, particularly stomatal regulation. However, leaf-level models sometimes assume infinite $g_{b}$, which can overlook aerodynamic limitation. These perspectives frame the exchange processes as either environmentally or biologically controlled, at different scales. In reality, both types of control operate simultaneously, highlighting the need for integrated models that account for multiple conductances shaped by both plant traits and environmental conditions (Table 1).

**Table 1.** Plant and environmental factors affecting heat and mass transport from the atmosphere to the chloroplast, and subsequent processes, through the aerodynamic conductance ($g_{a}$), and the conductances of the leaf boundary layer ($g_{b}$), the stomata ($g_{s}$), and the mesophyll ($g_{m}$)

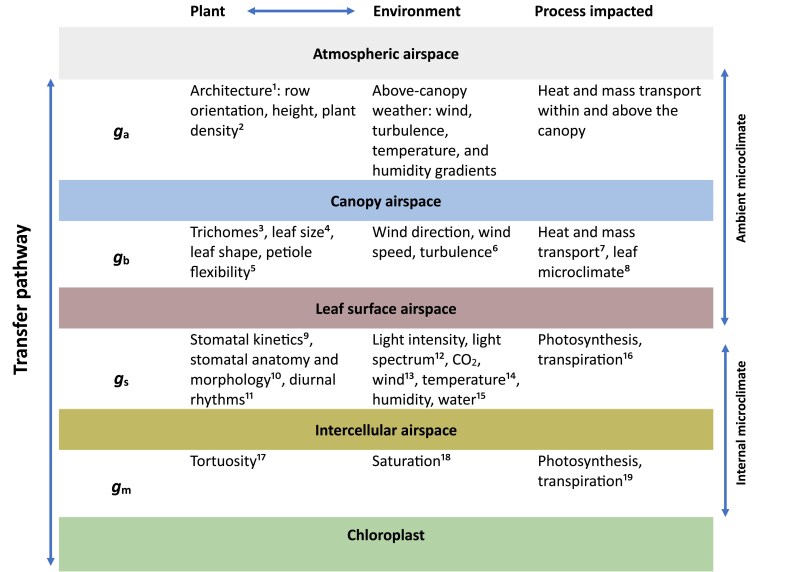

Cuticular conductance (*g*_c_) additionally impacts water exchange but is left out for simplicity (Márquez *et al*., 2022). The air in the atmosphere, inside the canopy, and around the leaf are part of the external ambient microclimate, while the intercellular airspace is the internal microclimate. References: ^1^(Meyers, 1989; Leuzinger and Körner, 2007); ^2^(Bailey *et al*., 2016); ^3^(Ehleringer and Mooney, 1978; Schuepp, 1993; Evans and Loreto, 2000; Saudreau *et al*., 2017; Galdon-Armero *et al*., 2018; Amada *et al*., 2023); ^4^(Leuzinger and Körner, 2007; Wright *et al*., 2017; Pan *et al*., 2022; Al-Salman *et al*., 2023); ^5^(Grace, 1978; Roden and Pearcy, 1993); ^6^(Brenner and Jarvis, 1995; Stokes *et al*., 2006); ^7^(Schuepp, 1993); ^8^(Aphalo and Jarvis, 1993; Savvides *et al*., 2013); ^9^(Faralli *et al*., 2019; Lawson and Vialet-Chabrand, 2019); ^10^(Franks and Farquhar, 2007; Al-Salman *et al*., 2023); ^11^(Matthews *et al*., 2018); ^12^(Matthews *et al*., 2020); ^13^(Carvalho *et al*., 2015); ^14^(Mills *et al*., 2024); ^15^(Buckley, 2019; Zhu *et al*., 2024); ^16^(Jarvis and McNaughton, 1986; Miner *et al*., 2017); ^17^(Harwood *et al*., 2021); ^18^(Wong *et al*., 2022); ^19^(Du *et al*., 2019).

### Transfer from atmosphere to canopy airspace

Mass and heat exchange between the atmosphere and plant canopy depend on the aerodynamic conductance (*g*_a_), which quantifies the ease with which gases and heat are transported from and to the atmosphere above the canopy to the canopy airspace due to turbulent air motion (Kim *et al*., 2014; Hirsch *et al*., 2016). Leaves rarely exist in aerodynamic isolation, and the canopy architecture generates aerodynamic drag, creating turbulence that slows the airflow and produces a shelter effect. This results in spatial and temporal microclimatic gradients of temperature, [H_2_O], and [CO_2_] within the canopy (Schuepp, 1972; Vilà-Guerau de Arellano *et al*., 2020). These gradients are shaped by radiation, plant gas exchange, and atmospheric concentrations above the canopy (Ney and Graf, 2018; Westreenen *et al*., 2020; Murchie and Burgess, 2022). Denser canopies or particular row orientations can further amplify aerodynamic resistance (Goudriaan, 1977; Campbell and Norman, 2000; Westreenen *et al*., 2020).

In field-grown crops, wind-driven mixing (1–8 m s^−1^) often minimizes these microclimatic gradients (Goudriaan, 1977; Desjardins *et al*., 1978; Baldocchi *et al*., 1983; Postma *et al*., 2021). However, under low-wind conditions (often at night), air within the canopy can decouple from the air above the canopy, inducing microclimatic gradients (Jacobs *et al*., 1995; van Gorsel *et al*., 2011). In contrast, CEA constantly exhibits substantial gradients in the within-canopy air due to poor air mixing (Majdoubi *et al*., 2009; Qian *et al*., 2009; Kimura *et al*., 2020a; Westreenen *et al*., 2020). Structural responses, such as thigmomorphogenesis triggered by sustained wind (typically >2 m s^−1^; Wadsworth, 1959; Jaffe, 1973; Smith and Ennos, 2003; Anten *et al*., 2010; Gardiner *et al*., 2016; Langer *et al*., 2022; Shibuya *et al*., 2022) affect *g_a_* and $g_{b}$ by altering the canopy architecture. This review focuses on leaf level processes involving $g_{b}$, but *g_a_* and *g_b_* act in series and influence the microclimate within the canopy. For a more comprehensive discussion on canopy-scale aerodynamic processes, readers are referred to Brunet (2020).

### Transfer from canopy airspace to chloroplasts

Gas exchange from the canopy microclimate to the chloroplasts occurs via diffusion involving three conductances in series: $g_{b}$, $g_{s}$, and $g_{m}$ (Table 1) (Evans and Loreto, 2000; Evans *et al*., 2009; Nobel, 2009). Beyond diffusion, convection (bulk air movement) affects heat and mass transfer around the leaf, influencing $g_{b}$ by altering the thickness of the boundary layer (Vogel, 2009). Free convection (buoyancy forces driven by temperature differences) and forced convection (fluid movement due to external forces such as wind) help refresh the air surrounding the leaf and can remove accumulated water vapour around the leaf from transpiration, which otherwise decreases $g_{b}$.

Mass and heat transport through the leaf boundary layer are substance-specific under laminar flow conditions, as diffusivities and thermal properties differ, resulting in different conductances for H_2_O ($g_{\mathrm{bw}}$), CO_2_ ($g_{\mathrm{bCO}2}$), and heat ($g_{\mathrm{bh}}$). These are often interconverted using diffusion coefficients, assuming equal path lengths in well-mixed conditions, although such differences diminish under turbulent flow where convection governs transport. For consistency, we express all numeric conductance values as $g_{\mathrm{bw}}\;$of one side of the leaf ($g_{\mathrm{bw},\mathrm{one}-\mathrm{sided}}$), and convert literature values accordingly to enable comparisons with $g_{s}$, which is typically expressed for H_2_O transfer. Additionally, $g_{\mathrm{bw}}$ is more widely reported than $g_{\mathrm{bCO}2}$, due to the majority of $g_{b}$ measurements being based on water or heat loss.

## Diffusive limitation by stomatal, mesophyll, and boundary layer conductances

The stomata are biologically controlled, responding within minutes to light, CO_2_, temperature, and humidity (Lawson and Blatt, 2014). The environmental signals (except for light) are sensed within the boundary layer, and are partially decoupled from the ambient air since stomata sense conditions next to the guard cells (Bunce, 1985; Peak and Mott, 2011). The responses are further modulated by plant-specific traits such as stomatal anatomy and patterning (e.g. density and pore size) (Table 1). These traits are shaped by environmental conditions during development, including evaporative demand (Carvalho *et al*., 2015; Bertolino *et al*., 2019; Du *et al*., 2019). Similarly, $g_{m}$ is determined by internal leaf anatomy (e.g. mesophyll surface area, porosity, tortuosity) and can acclimate to environmental factors such as high evaporative demand (Flexas *et al*., 2012; Cano *et al*., 2019; Harwood *et al*., 2021), which can cause unsaturation of the intercellular airspaces (Wong *et al*., 2022). These interactions show that the effectiveness of $g_{s}$ and $g_{m}$ depend in part on the aerodynamic coupling of the leaf. Hence, $g_{b}$ can impact photosynthesis by determining the microclimatic perception and acclimation responses of other conductances.

The boundary layer is primarily driven by environmental factors, namely wind speed and direction, and the intensity of turbulence, and by plant traits namely leaf size and shape, surface features (e.g. trichomes), and leaf-to-air temperature gradients (for reviews on leaf boundary layer physics, see Schuepp, 1993; Vogel, 2009). Notably, features such as trichomes and petiole flexibility can modulate $g_{b}$ in unclear or context-dependent ways. Trichomes can trap air, thereby thickening the boundary layer, or promote turbulence; leaf flutter can alter wind speed at the leaf surface, but also alter light capture, both of which influence photosynthesis (Ehleringer and Mooney, 1978; Grace, 1978; Roden and Pearcy, 1993; Schuepp, 1993; Evans and Loreto, 2000; Galdon-Armero *et al*., 2018; Pierce *et al*., 2022, Preprint). Natural variation in traits such as petiole flexibility, leaf shape, rolling, and surface microtopography is promising for modulating $g_{b}$ and might provide an opportunity to improve canopy photosynthesis or WUE, but they remain understudied and also simultaneously affect light interception (Drewry *et al*., 2014; Leigh *et al*., 2017; Cano *et al*., 2019; Pan *et al*., 2022; Al-Salman *et al*., 2023).

## Diversity and relative contribution of conductances to diffusive limitations

### Leaf boundary layer conductance: an underexplored limiting factor of photosynthesis?

For over 65 years, the boundary layer has been recognized as an important part of the transfer pathway (Gaastra, 1959). Jarvis and McNaughton (1986) expanded this understanding by providing a framework describing the control of transpiration by stomatal and boundary layer regulation from leaf to regional levels (several tens of kilometres). Despite this, the boundary layer is often excluded in analyses of photosynthesis limitation and optimization, under the assumption of high leaf-to-air coupling (Buckley *et al*., 2017; Kaiser *et al*., 2017; Deans *et al*., 2019), which has led to $g_{b}$ being historically viewed as less influential than $g_{s}$ or $g_{m}$. Its omission might also be partly due to challenges in accurate, spatially resolved measurement (Brenner and Jarvis, 1995; Stokes *et al*., 2006).

In windy field conditions, $g_{b}$ is often 10–100 times larger than $g_{s}$ (Jarvis and McNaughton, 1986; Martin *et al*., 1999; Nobel, 2009), rendering its contribution in limiting heat and gas exchange relatively minor, with limited impact on [CO_2_] gradients (Niinemets *et al*., 2009; Vico *et al*., 2013). This assumption is met in portable gas-exchange chambers where >2 m s^−1^ wind speeds lead to $g_{\mathrm{bw},\;\mathrm{one}-\mathrm{sided}}$ ranging from 1.25–5 mol H_2_O m^−2^ s^−1^, depending on leaf area, cuvette type, and fan speed, thereby minimizing its contribution to the transfer path length. However, this dynamic changes in low-wind environments, such as windless days, dense canopies, species with large leaves, and CEA with low air circulation rates. Under these conditions, the boundary layer effect is no longer negligible.

**Fig. 1.**
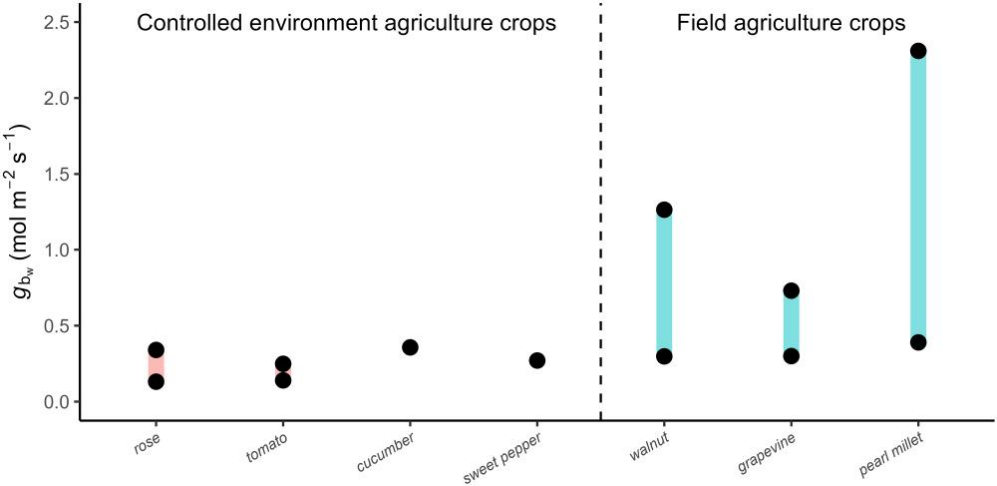
Ranges of reported values for one-sided boundary layer conductance to water vapour (*g*_bw_) in various controlled-environment agriculture and field crops. Values were measured with the heated-plate method using energy balance models. Heat-to-water conductance was converted by multiplying by 1.08206, assuming diffusion coefficients at 25 °C. Source of data: rose (*Rosa* sp.), Katsoulas *et al*. (2007); tomato (*Solanum lycopersicum*), Kimura *et al*., (2020b), J. Zhang *et al*. (2024); cucumber (*Cucumis sativus*) and sweet peper (*Capsicum annuum*), J. Zhang *et al*. (2024); walnut (*Juglans regia*) and grapevine (*Vitis vinifera*), Daudet *et al*. (1998); pearl millet (*Cenchrus americanus*), Brenner and Jarvis (1995).

In CEA, observations of $g_{\mathrm{bw},\;\mathrm{one}-\mathrm{sided}}$ fall between 0.13–0.36 mol H_2_O m^−2^ s^−1^ and in field crops between 0.27–2.31 mol H_2_O m^−2^ s^−1^ (Fig. 1). These ranges suggest that in CEA and sometimes in the field, $g_{\mathrm{bw}}\;$can be of similar magnitude to $g_{\mathrm{sw}}$ (Turner, 1991; N. Zhang *et al*., 2022). However, direct comparisons between CEA and field conditions for the same species are lacking, and most studies estimate $g_{\mathrm{bw}}\;$indirectly using wind speed and semi-empirical models. Hence, it is unlikely that the range presented in Fig. 1 is complete and more extreme scenarios might occur. We now further explore how diffusive limitations imposed by $g_{b}$ arise both temporally and spatially, driven by microclimatic gradients under low-wind conditions in CEA and field crops.

## Photosynthesis limitation by boundary layer conductance in controlled-environment agriculture

**Fig. 2.**
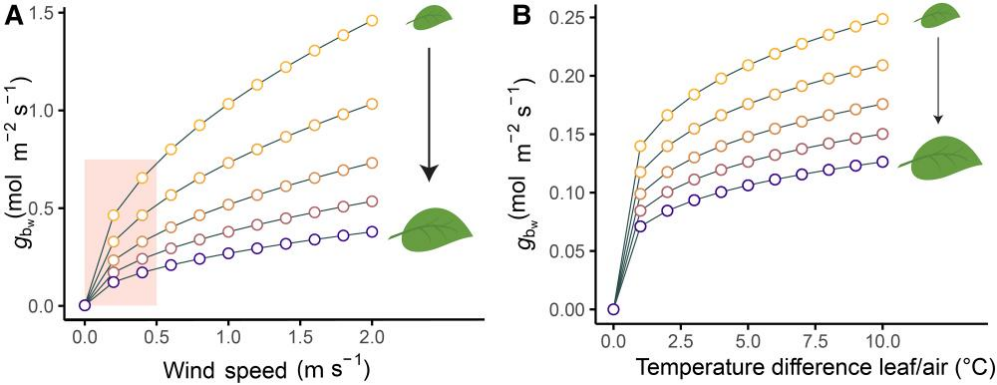
The responses of leaf boundary layer conductance to wind speed and leaf-to-air temperature differences for a variety of leaf sizes, based on semi-empirical physical models of heat transfer. (A) The responses of one-sided boundary layer conductance (*g*_bw_) to wind speed, assuming a leaf-to-air temperature difference of 0. *g*_b_ was calculated as $\left(0.664D^{2/3}u^{0.5})/(L^{0.5}v^{1/6}\right)$ (eqn. 1 in Brenner and Jarvis, 1995), and then converted to molar conductances using the ideal gas law, where *D* is the diffusion coefficient for heat or water (mm^2^ s^−1^), *u* is wind speed (m s^−1^), *v* is the kinematic viscosity of the air (mm^2^ s^−1;^ Appendix 2 in Jones, 2013), and *L* is the mean characteristic dimension of the leaf in the direction of the wind (Schuepp, 1993), in this case the length, which ranged from 0.02 m to 0.3 m. The shaded area shows the typical range of wind speeds for controlled-environment agriculture (Kimura *et al*., 2020b; Carpineti *et al*., 2024). Different variants of the basic formula exist depending on the assumptions made, such as the flow regime, and can be found in Schuepp (1993) and Brenner and Jarvis (1995) or ecophysiology books such as Nobel (2009), Jones (2013), and Hikosaka *et al*. (2016). (B) One-sided upper-surface boundary layer conductance to water vapour (g_bw_) in relation to the leaf-to-air temperature difference, assuming a wind speed of 0. Based on physical theory for free convection of heat transfer of a fixed flat plate, *g*_b_ was calculated as $\frac{0.54D^{0.75}G^{0.25}a_{t}^{0.25}{\left(T_{s}-T_{a}\right)}^{0.25}}{L^{0.25}v^{0.25}}$ (eqn. 3 in Brenner and Jarvis, 1995) and converted to molar conductances using the ideal gas law, where *G* is acceleration due to gravity (m s^−2^), *a*_t_ is the coefficient of thermal expansion of air (K^−1^) and $T_{s}-T_{a}$ is the temperature difference between the convective surface and the air.

Low wind speeds in CEA are a by-product of their enclosure and the costs required to reach high airflow, and they often drop to as low as 0.05 m s^−1^ (Katsoulas *et al*., 2007; Kimura *et al*., 2020b; Carpineti *et al*., 2024). Weak wind speeds (typically <1 m s^−1^) are often regarded as beneficial for faster growth in CEA. This is because low wind speeds lead to less mechanical stimulation, and can help prevent excessive transpiration and promote accelerated development due to higher temperatures at the meristem (Grace, 1988; Anten *et al*., 2010; Savvides *et al*., 2013). However, these conditions can also impede exchange processes and result in a non-homogeneous microclimate. In this low-wind range, a small increase can disproportionately raise the value of $g_{\mathrm{bw},\mathrm{one}-\mathrm{sided}}$, particularly below 0–0.5 m s^−1^ (shaded area in Fig. 2A). The larger the leaves, the less determining wind becomes, reducing the potential for short-term environmental manipulation of $g_{b}$.

Prediction of $g_{b}$ often assumes a laminar boundary layer under forced convection (Fig. 2A) and necessitates measurement of airflow (Box 1). Increasing wind speed increases the tendency of turbulence, and a laminar assumption will then underestimate $g_{b}$. Laminar boundary layers are still observed at wind speeds of 3.5 m s^−1^ for large leaves represented by realistically lobed replicas in tree canopies (Daudet *et al*., 1999; Stokes *et al*., 2006). These wind speeds are an order of magnitude higher than those encountered in CEA. The assumption of laminar flow in CEA is further supported by the reasonable agreement between laminar boundary layer predictions and measurements in greenhouses (Kimura *et al*., 2020b) (Fig. 2A versus Fig. 1). Another difficulty in predicting *g*_b_ that is often overlooked is how to measure the leaf characteristic dimension when leaves are overlapping or their orientation toward the air inlet is changing.

Box 1. Understanding and measuring airflow in controlled environment agriculture In contrast to other environmental variables, for airflow there is no consensus on the optimal placement of sensors near the plant or leaf, or on which characteristics should be measured, such as speed, direction, or turbulence. The International Committee on Controlled Environment Guidelines recommend reporting the design of the air circulation system, sensor type, predominant flow direction, the number of measurement points, and their location relative to the plant canopy (Both *et al*., 2015). Airflow sensors for CEA must detect low speeds (cm s^−1^), ideally capturing direction and turbulence, while being small enough to avoid disturbing the airflow. 3D sonic anemometers offer high accuracy and full directional data but are bulky; 2D sonics are smaller but less precise; hot-wire sensors struggle only with very low wind speeds (<0.1 m s^−1^) and are compact but lack directional data; and calorimetric sensors proved better low-speed accuracy than hot-wires but have a narrower dynamic range (Mauder *et al*., 2007; Lomas, 2011). Based on these characteristics, sonic sensors are suitable above or within canopies where space allows (e.g. between rows), while hot-wire and calorimetric probes are better suited for close-to-plant measurements. Microelectromechanical systems (MEMS) technology might improve sensor size and precision in the future (Ejeian *et al*., 2019), but capturing airflow near leaf surfaces remains difficult due to size and placement constraints such as overlapping leaves.

In near-zero wind conditions, free convection can be significant when leaf-to-air temperature differences are large (Fig. 2B). In greenhouses, this often leads to a mixed convection regime (Stanghellini, 1987, 1993; Grace, 1988; Brenner and Jarvis, 1995; Katsoulas *et al*., 2007; Vermeulen *et al*., 2012; Kimura *et al*., 2020b), which remains significant up to ∼0.6 m s^−1^, but rarely predominates (Vogel, 2009). When a mixed convection regime occurs, approaches to combine the free and forced convection have the following options: taking the maximum, summing in parallel, or using a smooth transition between the free and forced convection (Brenner and Jarvis, 1995; Leuning *et al*., 1995; Boekee *et al*., 2023). Given these difficulties, direct $g_{b}$ measurements are often preferable, as they inherently capture local flow conditions and plant morphology (Box 2).

Box 2. Novel methods for measuring leaf boundary layer conductance Measurements of $g_{b}$ are often preferable over estimates based on wind speed using semi-empirical formulas, as they inherently account for the effects of plant structure, leaf shape, position within the canopy, and nature of a variable airflow (i.e. turbulence and angle). Since *g*_b_ affects the heat and mass exchange between the leaf and surrounding air (see A in the figure), it can be determined by measuring temperature variations or water loss. Traditional methods, such as heated metal plates (Brenner and Jarvis, 1995; Jones, 2013; Vialet-Chabrand and Lawson, 2019; Kimura *et al*., 2020b) or wet filter papers (Gaastra, 1959), infer $g_{b}$ from cooling or evaporation rates, respectively. Heated plates allow automated measurement and are less prone to user error (e.g. inconsistent wetting of filter paper), but evaporative methods, despite requiring replenishment of water, are simpler to use, require minimal set-up and are closer to real leaves as they include mass flow. Both approaches rely on ‘leaf replicas’ that can be cut to match leaf shape (Stokes *et al*., 2006) and can be included in the canopy; however, they fail to capture the effect of the real leaf microstructure (e.g. trichomes and surface roughness) (Galdon-Armero *et al*., 2018). To overcome this, soft lithography can mimic the surface properties of the leaf (Soffe *et al*., 2019). More recently, transient energy balance methods have made use of transient changes in leaf temperature following a short light pulse (∼30 s) (J. Zhang *et al*., 2024), allowing spatially resolution of absorbed irradiance in whole plants. Leaf $g_{b}$ can be quantified while simultaneously considering energy storage in the leaf and its microstructure. Similarly to the heated plate method, a high $g_{b}$ by increasing wind accelerates cooling (see B in the figure), enabling spatial $g_{b}$ mapping of real leaves across the canopy.
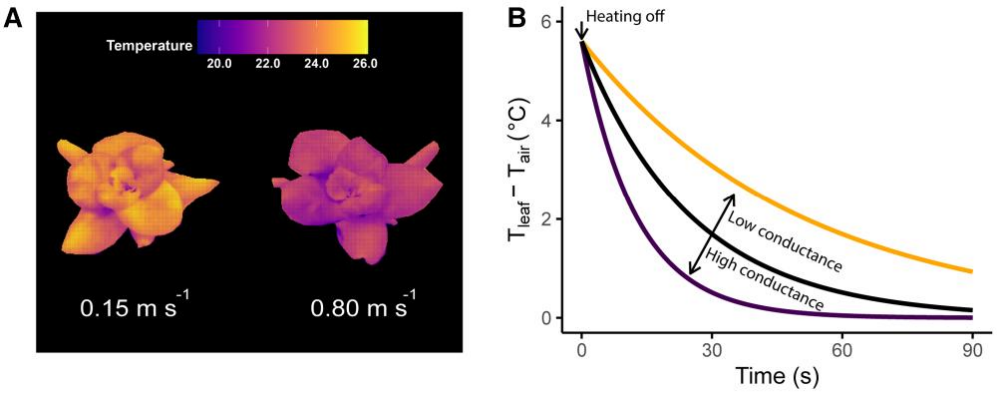
Impact of wind speed on leaf temperature and cool-down dynamics in lettuce plants (*Lactuca sativa*). (A) False-colour heatmap of leaf temperature (°C) under two different wind speeds at an air temperature of ∼21.1 °C. The plants were illuminated with a LED light source (Elixia; Heliospectra AB, Göteborg, Sweden) at 200 W m^−2^ for 5 min. Wind was generated using an electric fan and the lower speed was created by placing cardboard between the plants. (B) Cool-down of a replica leaf after a period of warming, expressed as the temperature difference between the leaf and the air. The cool-down curves are based on Newton’s law of cooling and assume that the energy exchange with the environment is primarily sensible heat transfer, after Appendix 8 in Jones (2013), estimated for an aluminium leaf model. The approach has also been used with real leaves (J. Zhang *et al*., 2024).

**Fig. 3.**
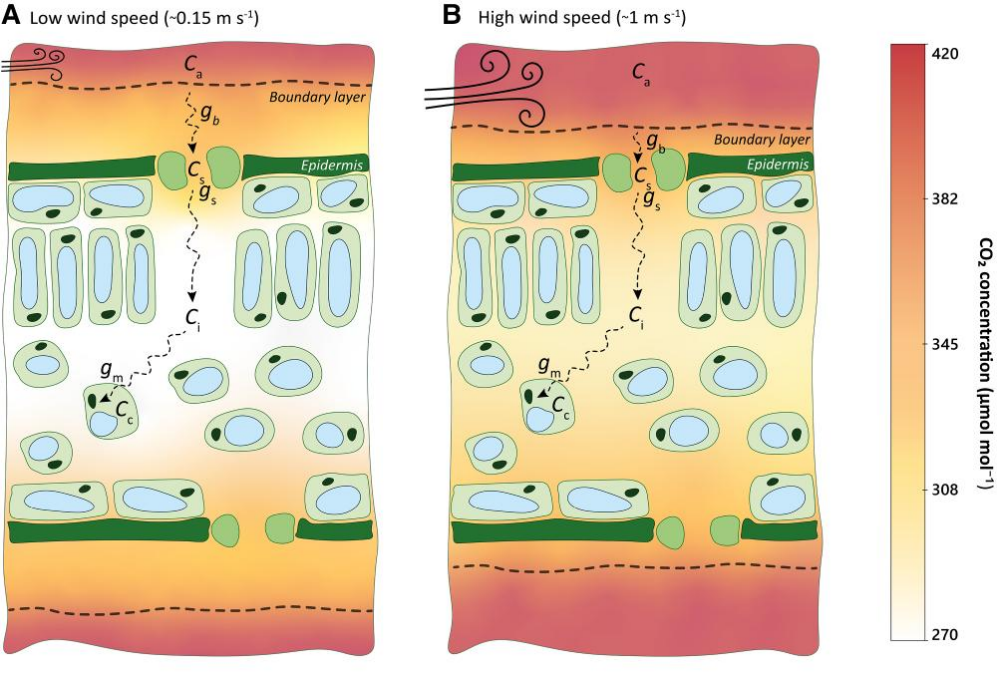
Schematic representation of CO_2_ diffusion through the leaf boundary layer and into the chloroplast under different wind speeds. The cross-sections represent a leaf with a uniform boundary layer and show the diffusional path of CO_2_ from the ambient air to the stomatal aperture and then to the chloroplast, under two different wind conditions. The corresponding distribution of [CO_2_] is shown, as indicated in the key. (A) A low wind speed results in a relatively thick boundary layer where [CO_2_] decreases from 420 µmol mol^−1^ in the ambient air ($C_{a}$), to 350 µmol mol^−1^ in the air at the stomatal aperture ($C_{s}$), to 270 µmol mol^−1^ in the intercellular airspace ($C_{i}$). (B) A higher wind speed results in a thinner boundary layer where *C*_s_ is decreased to 393 µmol mol^−1^ and *C*_i_ is decreased to 313 µmol mol^−1^. Intercellular and stomatal [CO_2_] were calculated using Fick’s law under typical climatic conditions (*C*_a_=420 µmol mol^−1^, 25 °C) and plant processes (*A*=20 µmol m^−2^ s^−1^, *g*ₛ=0.4 mol m^−2^ s^−1^), $C_{i}\;$was calculated as $C_{a}-A/[1/(1.6/g_{\mathrm{sw}}+1.37/g_{\mathrm{bw}})]$, and $C_{s}$ as $C_{a}-1.37\;\times A/g_{\mathrm{bw}}$, where *g*_sw_ and *g*_bw_ are respectively the stomatal and boundary-layer conductances to water vapour. The thickness of a laminar boundary layer for momentum transfer is proportional to the square-root of the distance along the airflow and the reciprocal of the free stream velocity (Schlichting and Gersten, 2016). However, here we visualize an average boundary layer thickness and ignore edge effects. The mean characteristic dimension of the leaf in the direction of the wind used in this example was 0.0825 m, which is an intermediate value, and resulted in values of one-sided $g_{\mathrm{bw}}\;$of 0.196 mol m^−2^ s^−1^ and 0.506 mol m^−2^ s^−1^ for the low- and high-wind speed scenarios, respectively (Brenner and Jarvis, 1995); these values were then multiplied by 2, assuming equally distributed stomata and equal contributions of heat and gas exchange of both surfaces of the leaf. The temperature of the leaf was assumed to be equal to that of the air and only forced convection was considered.

To illustrate the potential improvement of the CO_2_ supply chain in a greenhouse by manipulating $g_{b}$, two scenarios can be contrasted: one with low wind speed (0.15 m s^−1^) and thus low *g*_b_, and one with higher wind and thus higher *g*_b_ (1 m s^−1^; Fig. 3). Under low wind speed, the thick boundary layer depletes CO_2_ at the leaf surface, which further impacts [CO_2_] downstream in the intercellular airspace (Bunce, 1988; Aphalo and Jarvis, 1993). In this scenario, besides the stomata, the boundary layer substantially limits the CO_2_ supply chain: the boundary layer decreases [CO_2_] by 17% while the stomata decrease it by 23%. As a result, gradients of [CO_2_] in the canopy air become amplified at the leaf surface. Under high wind speeds, CO_2_ diffusion is enhanced, reducing [CO_2_] gradients between the leaf surface and canopy air. The boundary layer decreases [CO_2_] by 6% while the stomata decrease it by 20%.

### Finding an ideal boundary layer: revisiting the trade-off between CO_2_ uptake and H_2_O loss

Optimizing $g_{b}$ in the short-term (i.e. excluding development and acclimation) involves heat and gas exchange trade-offs depending on the environmental context and plant physiology. Wind-induced changes to the leaf microclimate trigger stomatal responses to intercellular [CO_2_], vapour pressure deficit at the stomatal aperture within the boundary layer ($D_{s}$; often also named as the deficit at the leaf surface) (Aphalo and Jarvis, 1993), and temperature (Mills *et al*., 2024). High wind typically reduces leaf surface humidity, prompting stomatal closure, although leaf cooling can mitigate this response (Schymanski and Or, 2016). In contrast, low wind increases leaf surface humidity, encouraging stomatal opening, yet a concurrent increase in leaf temperature will lead to a larger driving force of transpiration under intense radiation (Vogel, 2009; Leigh *et al*., 2012), and can also decrease photosynthesis if leaf temperatures deviate from the photosynthetic optimum (Collatz *et al*., 1991; Bernacchi *et al*., 2001; Yamori *et al*., 2014; Wright *et al*., 2017). These opposing effects underline the importance of the leaf energy balance.

Enhanced airflow in CEA can also mitigate issues such as tip burn through increased transpiration (Frantz *et al*., 2004), although at the cost of higher water use (Carvalho *et al*., 2015). The value of increasing $g_{b}$ is therefore context-dependent: it can improve CO_2_ uptake and thermal regulation under high radiation, but might offer limited gains under elevated ambient CO_2_ or in light-limited conditions (e.g. vertical farms). Differences in sensitivity to the environment and in plant traits (e.g. acclimation) can obscure the net effect of $g_{b}$ due to these interacting effects of CO_2_, water, and heat fluxes.

### Estimating the ideal boundary layer *in silico*

**Fig. 4.**
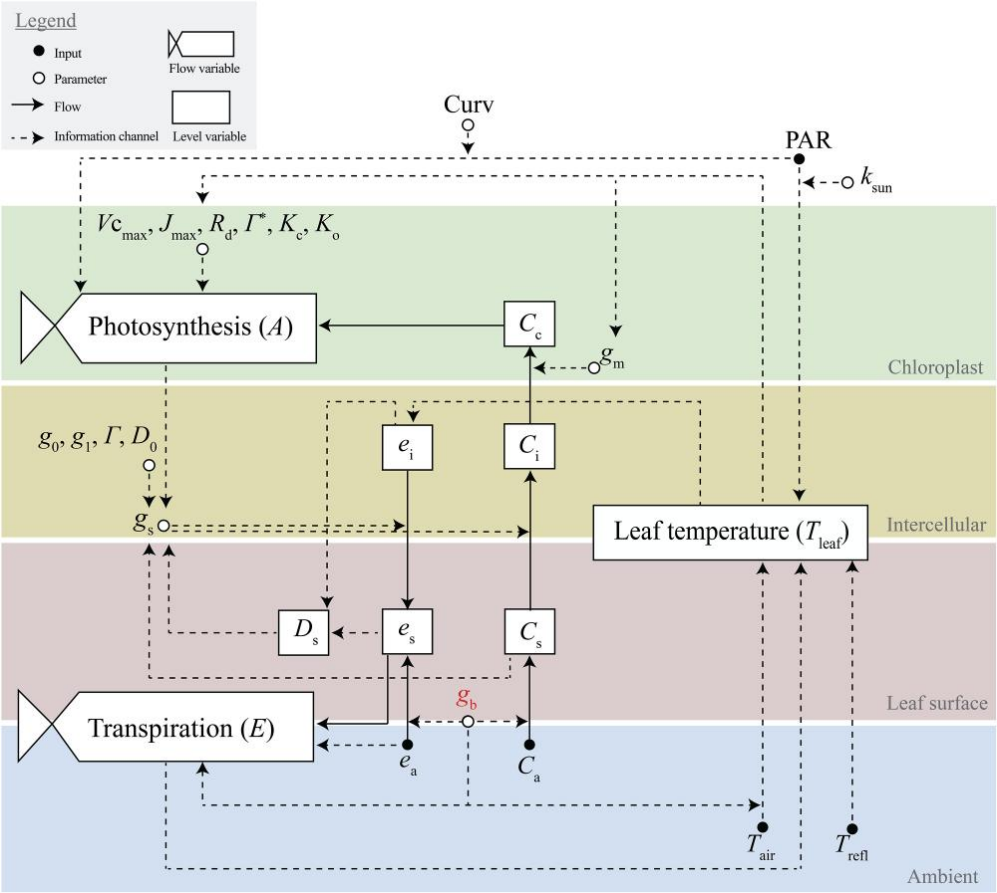
Simplified schematic representation of the leaf-level processes governing heat and gas exchange, integrating a photosynthesis model, an energy balance model, and a stomatal conductance model. The figure illustrates how CO_2_ and water vapour move through different compartments, from ambient air to the chloroplast and vice versa, as well as the key physiological parameters affecting these fluxes. The rates of photosynthesis and transpiration are influenced by environmental inputs, and the diffusion of CO_2_ is controlled by the conductances of the boundary layer ($g_{b}$), the stomata ($g_{s}$), and the mesophyll ($g_{m}$), while water vapour exchange is driven by vapour pressure gradients in the intercellular airspace ($e_{i}$), at the leaf surface ($e_{s}$), and in the surrounding air ($e_{a}$). The model incorporates photosynthetic and stomatal parameters to predict gas exchange responses to environmental conditions under different boundary layer conductances. Inputs (climatic variables) are represented with closed circles, while model parameters are shown with open circles. Flows, depicted as solid arrows, indicate movement of substances, whilst the flow variables describe the rate of change within the system. Level variables, such as those related to CO_2_ and H_2_O, represent concentrations that change over time due to inflows and outflows. Information channels, depicted as dashed arrows, indicate non-material influences such as feedback mechanisms and regulatory processes. Ambient pressure has been omitted from the inputs for clarity. Inputs: PAR, absorbed photosynthetically active radiation; *T*_air_, air temperature; *C*_a_, ambient CO_2_ concentration; *T*_refl_, reflected temperature. Concentrations and deficits: *C*_i_, intercellular CO_2_ concentration; *C*_s_, surface CO_2_ concentration; *e*_s_, surface H_2_O vapour pressure; *e*_a_, ambient H_2_O vapour pressure; *e*_i_, intercellular H_2_O vapour pressure, which assumes the leaf to be saturated; *D*_s_, leaf vapour pressure deficit at the surface. Conductances: *g*_m_, mesophyll conductance; *g*_s_, stomatal conductance; *g*_b_, boundary layer conductance. Photosynthetic parameters: *V*_Cmax_, maximum carboxylation velocity; *J*_max_, light-saturated potential rate of electron transport; *R*_d_, dark respiration rate; $\Gamma^{*}$ and *Γ*, CO_2_ compensation point in the absence and presence of $R_{d}$, respectively; *K*_c_ and *K*o, Michaelis constant for CO_2_ and O_2_, respectively; Curv, curvature of the relationship *J* versus *PAR*. Stomatal parameters: *g*_0_, nocturnal stomatal conductance; *g*_1_, slope of A/$g_{s}$; *D*_0_ sensitivity factor for the $D_{s}$ response. Others: *k*_sun_ conversion factor from PAR to W m^−2^.

An ideal boundary layer balances CO_2_ uptake, water conservation, and thermal regulation, and varies with environmental and crop-specific factors. Leaf heat and gas exchange models, such as those by Collatz *et al*. (1991) and Aphalo and Jarvis (1993), provide a tool to illustrate the complex trade-offs and short-term interactions induced by variations in *g*_b_. These models incorporate a boundary layer to calculate environmental conditions at the leaf surface (within the boundary layer), rather than relying only on ambient air conditions. In the following section, we show how to build and use such a model based on existing models. We adopt the approach established by Collatz *et al*. (1991), who combined a leaf photosynthesis model, a Ball–Berry-type stomatal model parameterized at the leaf surface, and an energy-balance model (Fig. 4).

#### Leaf photosynthesis model.

The Farquhar–von Caemmerer–Berry (FvCB) model (Farquhar *et al*., 1980; Von Caemmerer, 2013) was used to model net CO_2_ assimilation (*A*), determined as the minimum of the Rubisco-limited carboxylation rate ($A_{c}$) and the electron-transport limited rate ($A_{j}$). Limitation by triose phosphate utilization was not included, as it becomes influential mainly under low photorespiratory conditions (Ellsworth *et al*., 2015). In contrast to Collatz *et al*. (1991), the smoothing transition between the limiting rates was excluded due to limited mechanistic support (Walker *et al*., 2021; Van Diepen *et al*., 2022). $A_{c}$ and $A_{j}$ were calculated according to Ethier and Livingston (2004) with a non-rectangular hyperbola formulation to include internal CO_2_ transfer resistance. To simulate diurnally, a simple exponential induction and relaxation of *A* was implemented to include non-steady states (Mott and Woodrow, 2000; Vialet-Chabrand *et al*., 2021), making it possible to use this model under fluctuating environmental conditions (e.g. light intensity):


(1) $$\frac{dA}{dt}=\frac{A^{*}-A}{\tau_{A}}$$

where *A* is the instantaneous value, $A^{*}$ is the steady-state target, and *τ_A_* is the time constant to reach 63% of the total variation, with different time constants for an increase or decrease. To predict over a wide range of temperatures (10–40 °C), the photosynthesis parameters were temperature adjusted (Bernacchi *et al*., 2001, 2002, 2003; Sharkey *et al*., 2007).

#### Leaf energy balance and temperature.

Leaf temperature was calculated following Vialet-Chabrand and Lawson (2019) using an energy balance approach that accounts for longwave radiation exchange ($H_{\mathrm{lw}}$), shortwave radiation absorption ($H_{\mathrm{sw}}$), and convective and latent heat fluxes ($H_{\mathrm{conv}},\;\;H_{\mathrm{latent}}$), thereby accounting for the impact of the boundary layer on leaf temperature:


(2) $$\frac{dT}{dt}=\frac{H_{\mathrm{lw}}+H_{\mathrm{sw}}-H_{\mathrm{conv}}-H_{\mathrm{latent}}}{k}$$

*k* is the amount of energy per unit required to change the temperature of the material.

#### Stomatal conductance model.

$g_{s}$ was modelled using the Leuning (1995) formulation, an adaptation of the Ball–Berry model that accounts for CO_2_ drawdown and vapour pressure deficit effects at the stomata, and includes the minimal conductance as the nocturnal conductance ($g_{0}$), the slope of *A*/$g_{s}$ ($g_{1}$), a sensitivity factor for the $D_{s}$ response ($D_{0}$), [CO_2_] at the stomatal aperture ($C_{s}$), and the CO_2_ compensation point (*Γ*).


(3) $$g_{s}=g_{0}+g_{1}\frac{A}{C_{s}-\Gamma }\frac{1}{1+D_{s}/D_{0}}$$

An exponential induction and relaxation of $g_{s}$ was implemented:


(4) $$\frac{dg_{s}}{dt}=\frac{g_{s}^{*}-g_{s}}{\tau_{g_{s}}}$$

where $g_{s}$ is the instantaneous value, $g_{s}^{*}$ the steady-state target, and $\tau_{g_{s}}$ is the time constant to reach 63% of the total variation.

#### Transport model.

The CO_2_ diffusion across different compartments was described by including the CO_2_ concentration of the ambient or canopy air ($C_{a}$), at the stomatal aperture ($C_{s}$), in the intercellular airspace ($C_{i}$) and at the chloroplast ($C_{c}$). The diffusion between these compartments depends on the conductances of the boundary layer ($g_{b}$), stomata ($g_{s}$), and mesophyll ($g_{m}$), respectively. This leads to three conductances contributing to the total conductance to CO_2_ diffusion and determines $C_{c}$ as:


(5) $$C_{c}=\;C_{a}-A\left(\frac{1}{g_{b}}+\frac{1}{g_{s}}+\frac{1}{g_{m}}\right)$$

Notably, the [CO_2_] at the stomata differs from the concentration in the air due to $g_{b}$:


(6) $$C_{s}=\;C_{a}-\frac{A}{g_{b}}$$

The outward water vapour diffusion flux (*E*) was calculated from the vapour pressure gradient from the intercellular airspace ($e_{i}$) to the ambient air ($e_{a}$) and the total conductance to water vapour:


(7) $$E=\left(\frac{1}{\left(\frac{1}{g_{b}}+\frac{1}{g_{s}}\right)}\right)(e_{i}-e_{a})$$

To correct boundary layer effects on vapour pressure deficit, the Aphalo and Jarvis (1993) correction was applied to calculate a vapour pressure deficit at the stomatal aperture:


(8) $$D_{s}=(e_{i}-e_{a})\;(1-\;g_{t}/g_{b})$$

where *g*_t_ is the total conductance to water vapour in Eqn 7 above, 1/[(1/*g*_b_)+(1/*g*_s_)]. See Fig. 4 for full list of model parameters and abbreviations.

The gas exchange simulations that follow below assume that leaves are amphistomatous with equal stomatal distribution and the adaxial and abaxial surface flux contributions are equal. Consequently, in the calculations $g_{b,\mathrm{one}-\mathrm{sided}}$ is multiplied by 2 to represent the total $g_{b}$ for both surfaces in parallel. While this simplification rarely holds across species and environmental conditions (Foster and Smith, 1986; Muir, 2015; Wall *et al*., 2022), it allows us to focus on the underlying processes and to illustrate the impact of $g_{b}$ under idealized conditions. In reality, combining surface-specific $g_{s}$ and $g_{b}$ complicates the flux calculations (Guilioni *et al*., 2008; Muir, 2015), and accurate modelling of fluxes from the adaxial and abaxial surface will become more feasible as data on surface-specific gas exchange and conductance become more available (Márquez *et al*., 2022; Wall *et al*., 2022).

#### Simulation approach of the combined model.

Two complementary scenarios are modelled and compared with existing simulations, each parametrized with values from literature, namely a diurnal greenhouse simulation for tomato with a variety of constant $g_{b}$ values, and a steady-state profile simulation for field-grown wheat with a declining $g_{b}$ profile and vertical microclimate profile. This approach is based on several considerations. CEA has low but relatively stable airflow, supporting constant $g_{b}$ in a diurnal simulation. Moreover, the limited availability of data on the spatial variability of $g_{b}$ (or wind) in greenhouses necessitates the use of a constant value at a given height. A diurnal simulation is also particularly relevant for CEA because airflow can be actively controlled (e.g. through fans), whereas in the field $g_{b}$ is largely determined by external wind conditions. In field crops, low airflow is primarily a within-canopy phenomenon, making vertical profiles more relevant and distinct from CEA. While both simulation types could be applied to either system, this avoids redundancy and allows the conclusions from both simulations to be complementary. The conclusions drawn from the models apply to any crop growing under conditions where low wind speed occurs, only the magnitude of the response changes based on the leaf properties.

For appropriate $g_{b}$ input, the diurnal greenhouse tomato simulation assumes values within the range of reported CEA measurements and in response to enhanced airflow scenarios (Kimura *et al*., 2020b; J. Zhang *et al*., 2024). In contrast, $g_{b}$ for the wheat simulation was predicted with wind speed as input and a variety of leaf sizes, using the theoretical relationship shown in Fig. 2A, under the assumption of forced convection and a laminar boundary layer.

### Temporal dynamics of diffusive limitations of photosynthesis in controlled environment agriculture

**Fig. 5.**
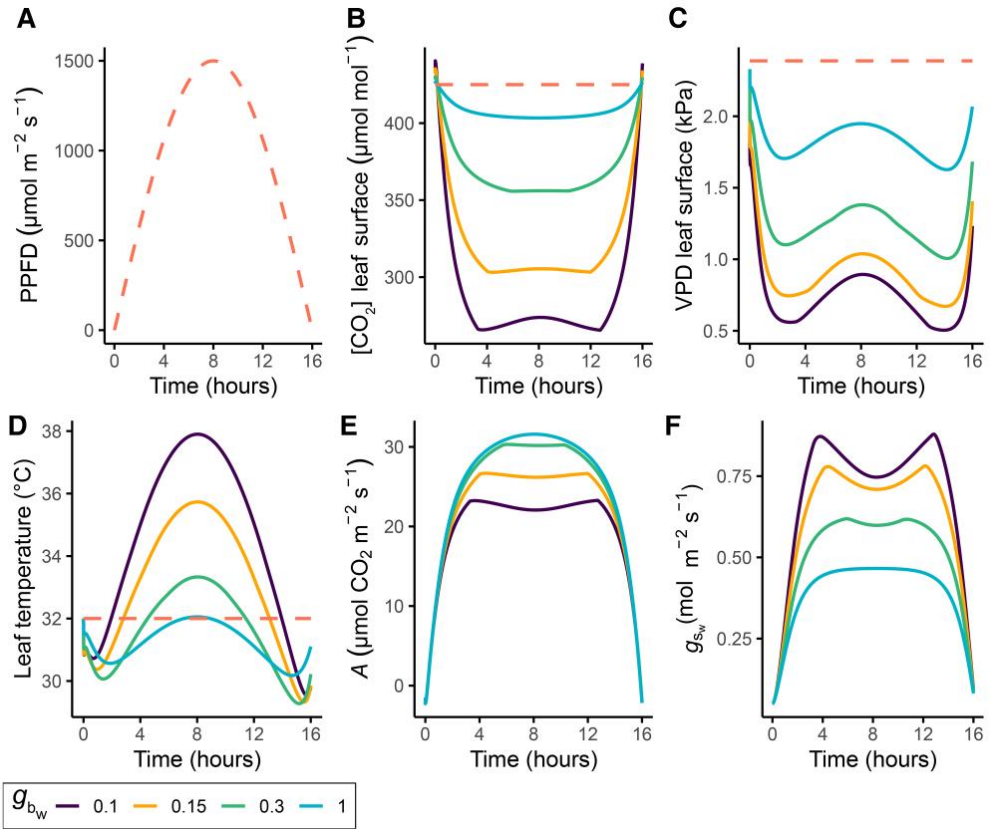
Simulated gas exchange and energy balance of a tomato leaf during a 16 h photoperiod under a variety of boundary layer conductances. The dashed lines represent the canopy environment, whilst the solid lines indicate different levels of one-sided boundary layer conductance to water vapour (*g*_bw_) in the range 0.1–1 mol H_2_O m^−2^ s^−1^, as shown in the key. The value for one side of the leaf was calculated and then multiplied by 2 to account for both sides. The results were obtained by simulating a tomato leaf using the combined Farquhar–von Caemmerer–Berry biochemical photosynthesis, stomatal conductance, and energy balance model. Bulk transport of the leaf was modelled using the approach of Collatz *et al*. (1991), assuming symmetrically amphistomatous leaves for tomato (N. Zhang *et al*., 2022). (A) Incident photosynthetically active radiation shown as photosynthetic photon flux density with a maximum of 1500 μmol m^−2^ s^−1^. (B) CO_2_ concentration at the leaf surface; ambient air is 425 μmol mol^−1^. (C) Vapour pressure deficit (VPD; kPa) at the leaf surface ($D_{s}$), calculated using the leaf VPD, the total conductance, and the boundary layer conductance: ($e_{i}-e_{a})\;(1-\;g_{t}/g_{b})\;$(Aphalo and Jarvis, 1993), where *e*_i_ is the intercellular H_2_O vapour pressure, *e*_a_ is the ambient H_2_O vapour pressure (2.4 kPa, or 50%), *g*_t_ is the total conductance to water vapour, and *g*_b_ is the conductance of the boundary layer. (D) Leaf temperature at an ambient temperature of 32 °C. (E) Net photosynthetic rate (*A*). (F) $g_{s}$ Stomatal conductance to water vapour (*g*_sw_). Typical parameters for tomato were taken from Y. Zhang *et al*. (2022) and Y. Zhang *et al*. (2024) unless otherwise stated, as follows. *V*_cmax25_=141 μmol CO_2_ m^−2^ s^−1^, where ‘25’ indicates standardization to a temperature of 25 °C; *J*_max25_=186 μmol CO_2_ m^−2^ s^−1^; *g*_m25_=3.2 μmol m^−2^ s^−^1 Pa^−1^; *R*_d25_=1.5 μmol CO_2_ m^−2^ s^−1^ (with temperature corrections from Sharkey *et al*., 2007); Abs_PAR_=0.84; Abs_shortwave_=0.50; Curvature of the relationship *J* versus *PAR*=0.70; time constant to reach 63% of total variation of *A* when increasing, $\tau_{Ai\;}$=300 s, and time constant to reach 63% when decreasing, $\tau_{Ad\;}$=1 s (Vialet-Chabrand *et al*., 2021); *k*_sun_ conversion factor from μmol m^−2^ s^−1^ to W m^−2^, calculated as $\frac{1}{\frac{500}{120}\times 0.47}$ (Stanghellini *et al*., 2019); nocturnal stomatal conductance, *g*_0_=0.05 mol m^−2^ s^−1^; slope of *A*/*g*_s_, *g*_1_=11 (McAusland *et al*., 2016); sensitivity factor for$\;D_{s}$ response, *D*_0_=1.5 (Leuning, 1995); time constant to reach 63% of total variation of $g_{s}$ when increasing, $\tau_{gi\;}$=900 s, and time constant to reach 63% when decreasing, $\tau_{gd}$=450 s (Vialet-Chabrand *et al*., 2021).

The model parametrized with $g_{\mathrm{bw}}$ values of 0.10, 0.15, 0.30, and 1 mol m^−2^ s^−1^ (*g*_bw,one-sided_) and constant environmental conditions except for light (Fig. 5A) illustrates heat and gas exchange interactions. Increasing $g_{\mathrm{bw}}\;$on both leaf surfaces from 0.10 mol m^−2^ s^−1^ to 0.15 mol m^−2^ s^−1^ leads to an increase of 13% in the daily integrated net photosynthesis rate (Fig. 5E), which could be achieved by increasing wind speed by only 0.05 m s^−1^ (Fig. 2A), assuming a characteristic leaf dimension (*L*) of either 0.05 m or 0.1 m. A further increase in $g_{\mathrm{bw}}$ from 0.15 mol m^−2^ s^−1^ to 0.30 mol m^−2^ s^−1^, which can be achieved by increasing wind speed by another 0.15–0.30 m s^−1^ (depending on leaf size), leads to an additional 10% increase in photosynthesis. These gains result from increased [CO_2_] at the leaf surface (Fig. 5B), which increases intercellular [CO_2_] (not shown) and outweighs the effect of stomatal closure (Fig. 5F), although this might vary by species (Schymanski and Or, 2016).

A low $g_{\mathrm{bw}}$ decreases the vapour pressure deficit ($D_{s}$) by increasing leaf surface humidity (Fig. 5C) while raising leaf temperature (Fig. 5D). At very low $g_{\mathrm{bw}}$ (0.10 mol m^−2^ s^−1^) photosynthesis is inhibited at midday due to high leaf temperatures (Fig. 5D, E), as previously pointed out by Collatz *et al*. (1991). Midday declines can also arise at high $g_{bw}$ values (2 mol m^−2^ s^−1^) due to the triggering stomatal closure by decreased leaf surface humidity (Collatz *et al*., 1991). While such high $g_{b}$ conditions are less relevant for CEA, these midday declines are consistent with field experiments showing similar patterns in leaf photosynthesis and stomatal conductance (Pan *et al*., 2022), where $g_{b}$-driven changes in leaf temperature play a substantial role (Kimura *et al*., 2024).

These simulations highlight that the perception of the canopy environment by the leaf varies, and depending on the leaf-surface microclimate and physiology, the response will differ greatly. This leads to significantly different leaf heat and gas exchange kinetics and further illustrates the interaction between *g*_b_ and temporal variation in climatic factors (here only light).

### Dynamic adjustments of the boundary layer can alleviate photosynthetic limitations

**Fig. 6.**
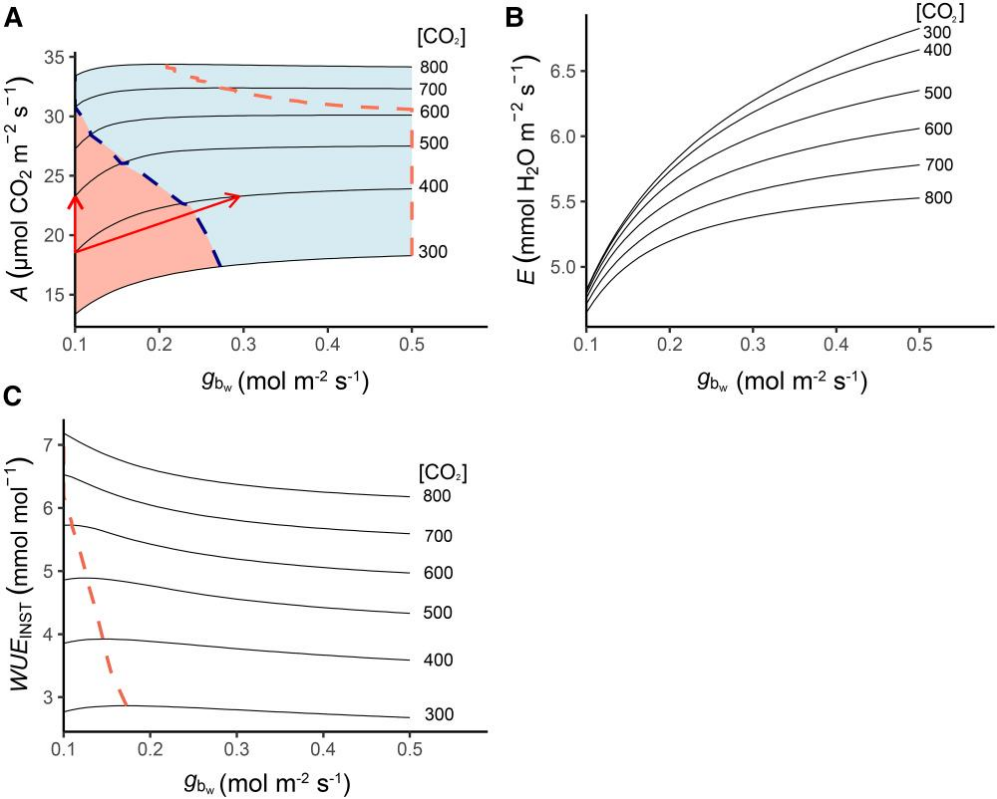
Simulated mean daily values of gas and heat exchange of a tomato leaf under varying [CO_2_] and boundary layer conductance conditions. The combined Farquhar–von Caemmerer–Berry biochemical photosynthesis, stomatal conductance, and energy balance model was used under combinations of ambient [CO_2_] ranging from 300–800 μmol mol^−1^ and one-sided boundary layer conductance to water vapour (*g*_bw_) ranging from 0.1–0.5 mol m^−2^ s^−1^. Other parameters are as listed in Fig. 5. (A) mean daily net photosynthesis rate (*A*). The red dashed line depicts the maximal rate for each [CO_2_] and the blue dashed line indicates 95% of the maximal rate. The red arrows indicate that a similar increase in *A* can be obtained by increasing [CO_2_] from 400 μmol mol^−1^ to 500 μmol mol^−1^, or by increasing *g*_bw_ from 0.1 mol m^−2^ s^−1^ to 0.3 mol m^−2^ s^−1^. (B) Mean daily transpiration rate (*E*). (C) Mean daily instantaneous water use efficiency ($WUE_{\mathrm{INST}}$). The dashed line depicts the maximal rate for each [CO_2_].

Simulations with the same parametrization were conducted to obtain mean net photosynthetic rates, transpiration rates, and instantaneous water-use efficiency ($WUE_{\mathrm{INST}}$), across a range of [CO_2_] (300–800 μmol m^−2^ s^−1^; Fig. 6). The lower concentrations represent depletions in naturally ventilated greenhouses without enrichment, while the higher concentrations represent CO_2_-enriched greenhouses (Qian *et al*., 2012). As CO_2_ enrichment will be reduced in the future (Zhou *et al*., 2021), the simulations assessed whether manipulating $g_{\mathrm{bw}}$ could compensate for reduced CO_2_ availability. At low [CO_2_], higher $g_{\mathrm{bw}}$ maximizes photosynthesis, but as [CO_2_] increases, this maximum shifts towards lower $g_{\mathrm{bw}}$ values (red dashed line in Fig. 6A). As a target for improvement, 95% of this maximum (blue dashed line) could minimize boundary layer limitations. In greenhouses with low $g_{\mathrm{bw}}$ (e.g. 0.10 mol H_2_O m^−2^ s^−1^, one-sided), enhancing air circulation to increase it could result in similar photosynthetic improvements to CO_2_ enrichment (red arrows). These results illustrate that an ‘optimal’ boundary layer strongly depends on the surrounding environmental conditions and that dynamic airflow control could serve as an effective tool for climate manipulation and resource optimization. While this example varies only CO_2_ and $g_{\mathrm{bw}}$, future extensions could incorporate temperature, light, and humidity. An interactive version of this model is available at www.plantgasexchange.com.

### The boundary layer as a modulator of WUE in controlled environment agriculture

A high $g_{b}$ can come at the cost of increased transpiration and reduced $WUE_{\mathrm{INST}}$ (Fig. 6B, C), since the total conductance to water vapour (*g*_t_) can increase, even if *g*_sw_ decreases. However, a high $g_{b}$ can also enhance WUE, depending on the interaction between diffusion gradients, radiation, temperature, and water availability (Parkhurst and Loucks, 1972; Leigh *et al*., 2012, 2017; Carvalho *et al*., 2015; Schymanski and Or, 2016; Wright *et al*., 2017; McAusland *et al*., 2021; Pan *et al*., 2022). This interplay is reflected in the Bowen ratio, which describes how surface energy is partitioned between heating the air (sensible heat) and evaporating water through soil evaporation and plant transpiration (latent heat). A low Bowen ratio (<1) typically reflects a well-watered crop, where most energy drives evapotranspiration. A high Bowen ratio (>1) suggests dry conditions, with stomata more closed, where limited moisture reduces latent heat flux, and more energy heats the air. Under high Bowen ratios, high $g_{b}$ tends to enhance sensible heat exchange, cooling leaf surfaces via convection and reducing transpiration. Under low Bowen ratios, increased $g_{b}$ can increase transpiration due to high latent heat exchange. Therefore, the Bowen ratio helps contextualize how $g_{b}$ influences the feedback between evaporative and convective cooling, and can indicate a range of $g_{s}$ where transpiration might decrease with increasing wind (Huang *et al*., 2015; Schymanski and Or, 2016).

Additionally, some factors were not accounted for in this model and could therefore lead to an overestimation of transpiration of high compared to low $g_{b}$. Water vapour build-up in enclosed spaces lowers the diffusion gradient for transpiration (Westreenen *et al*., 2020) and was not included. Unlike a constant-temperature assumption, air temperatures rise with light intensity, increasing leaf temperatures (especially for low $g_{b}$) more than in this modelled scenario.

## Photosynthesis limitation by boundary layer conductance in the field

### Predicting the boundary layer under fluctuating flow regimes

Estimating $g_{b}$ in the field is more challenging than in CEA due to fluctuating wind, variable leaf orientation, wind angle, and canopy turbulence, which alter the effective leaf dimension and leaf boundary layer properties (Nilsen and Forseth, 2018; Boekee *et al*., 2023). While laminar leaf boundary layers are a reasonable assumption under low airflow (Fig. 2A), outdoor environments involve transitions between laminar and turbulent flow (Brenner and Jarvis, 1995).

In flat-plate theory, the laminar–turbulent transition occurs around 30 m s^−1^ (*L*=0.05 m), when the air above the plate (free stream) is predominantly laminar (Monteith and Unsworth, 2013). When the air above is turbulent, the transition occurs at lower velocities (Jones, 2013). In nature, for leaves with structural features such as trichomes, transitions can begin as low as 0.7–1.35 m s^−1^ (Grace and Wilson, 1976; Grace, 1978; Brenner and Jarvis, 1995), based on rapid wind-speed fluctuations measured within the leaf boundary layer. Differences in trichomes can introduce species-specific and within-species differences (Baldocchi *et al*., 1983) in the laminar–turbulent transition that are further determined by the growth environment (Galdon-Armero *et al*., 2018).

To account for turbulence, some models apply correction factors (e.g. ×1.5; Jones, 2013), while others retain the laminar assumption (Monteith and Unsworth, 2013). Free convection, driven by leaf-to-air temperature gradients, can also contribute, for example for sunlit leaves within canopies (Leuning *et al*., 1995), but its prevalence is poorly quantified, although it is known that large fluctuations in light intensity within the canopy occur (Burgess *et al*., 2021). Directly measuring $g_{b}$ on leaves (Box 2) provides an ‘apparent’ $g_{b}$ that includes all these effects. For simplicity, the following simulations assume laminar boundary layers under forced convection, an approach supported by heated-plate measurements at low field wind speeds (Brenner and Jarvis, 1995; Stokes *et al*., 2006).

### Vertical wind and boundary layer conductance profiles in field crop canopies

Canopy microclimates are highly heterogeneous, with gradients in light intensity, light spectrum, humidity, wind speed, and other factors (Burgess *et al*., 2015; Vilà-Guerau de Arellano *et al*., 2020; Westreenen *et al*., 2020). These environmental gradients not only vary vertically but also horizontally, with exacerbated effects in CEA (Teitel *et al*., 2010). After analysing how $g_{b}$ limits leaf gas exchange, it becomes crucial to understand its spatiotemporal variations in the field.

**Fig. 7.**
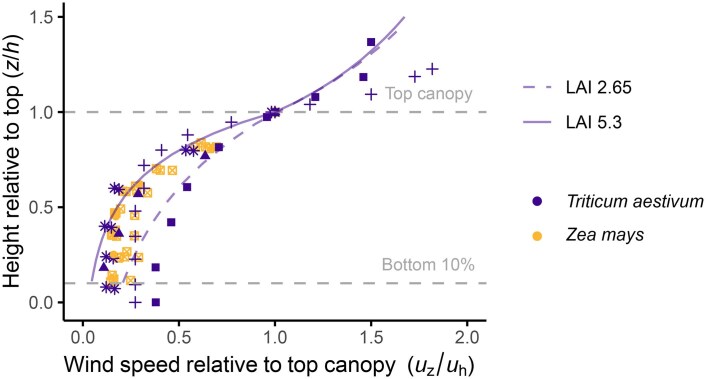
Measured and predicted wind profiles within crop canopies for *Triticum aestivum* (common wheat) and *Zea mays* (maize), with modelled wind speed predictions at different values of leaf area index (LAI). For relative height, *h* is the canopy height and *z* is the height within the canopy. Symbols represent measured wind speeds with data from: +, ▮, Vilà-Guerau de Arellano *et al*. (2020); ⊠, Desjardins *et al*. (1978); ▴, Cionco (1978); *, Legg and Long (1975); and ●, Businger (1975). Predicted wind profiles for wheat with LAIs of 5.30 and 2.65 are shown. The wind-speed decline above the top of the canopy (relative height >1) was calculated according to Eqn. 5.1 from Campbell and Norman (2000): *u_z_*=(u*/0.4)ln[(*z–d)*/*z*_m_]. u* is the friction velocity, which is constant and obtained by re-arranging the equation using the height of the anemometer. The zero plane displacement (*d*) was calculated as 0.7h with 0.45 m height chosen as representative for a crop with LAI 5.30 (Burgess *et al*., 2015) and as 0.65h with 0.24 m height for a crop with LAI 2.65. The roughness length ($z_{m}$) was calculated as 0.07h for LAI 5.30 and as 0.1h for LAI 2.65. The decline within the canopy was calculated according to Eqn. 5.4 from Campbell and Norman (2000): *u_z_*=*u*_top_exp[*a*(*z*/*h*–1)] , where the attenuation coefficient *a* was calculated as $(0.2LAIh/m{)}^{0.5}$ (Goudriaan, 1977) and the mean distance between the leaves *m* as ($4wh/\pi LAI{)}^{0.5}$, where *w* is the leaf width. Wind profiles are assumed to be valid up to a height (*z*) of 0.01 × fetch, where fetch is the surface over which wind has blown, for example the distance downwind from the edge of a field (Campbell and Norman, 2000; Jones, 2013).

A wind profile above the field can be predicted using the wind speed measured above the canopy at a reference height and the roughness characteristics of the crop (for details see Campbell and Norman, 2000). The vertical decline within the canopy can be calculated with an attenuation coefficient depending on the crop architecture. A denser crop with higher leaf area index causes a steeper decline in wind speed (Fig. 7). Similar equations for smaller canopies (e.g. in a vertical farm or greenhouse) with unnatural flow regimes do not exist yet, making it difficult to know what wind speed is perceived by the leaf when using forced air circulation such as fans.

### Temporal dynamics of the boundary layer in the field

**Fig. 8.**
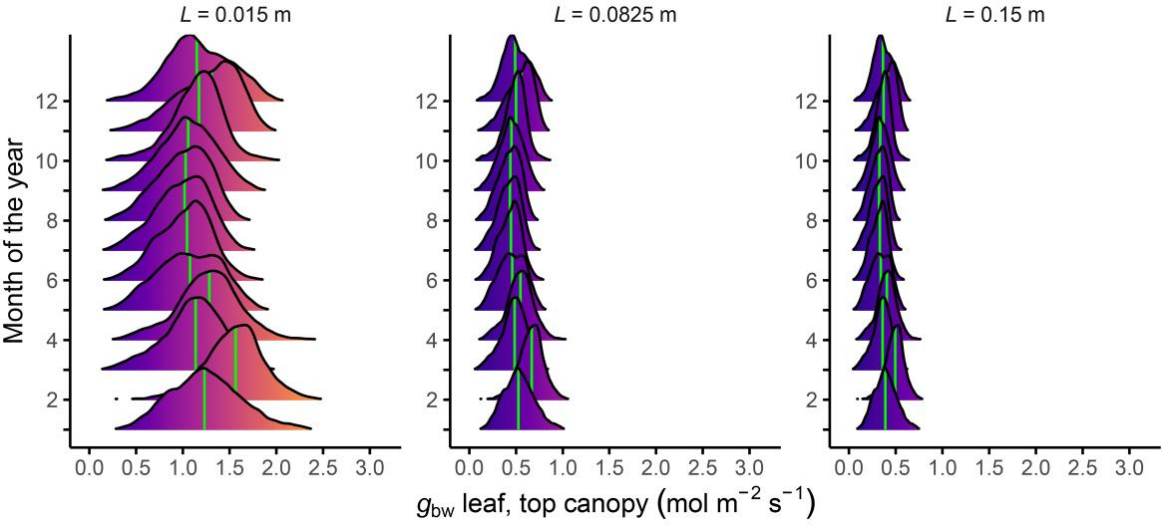
Annual variation in the distribution of predicted one-sided daytime boundary layer conductance for water vapour (*g*_bw_) for leaves at the top of the canopy that differ in their characteristic leaf dimension (*L*). A laminar boundary layer was assumed and *L* represents the mean length in the direction of the wind. The wind speed at 2 m height above a grass field was measured with a sonic anemometer (1 min averages), and was recalculated for the top of a winter wheat canopy using Eqn. 5.1 in Campbell and Norman (2000): *u_z_*=(u*/0.4) ln[(*z–d)*/*z*_m_] . The zero plane displacement (*d*) was calculated as 0.7h with 0.475 m height chosen as representative for a crop with LAI 5.30 (Burgess *et al*., 2015), the roughness length ($z_{m}$) calculated as 0.1h and the friction velocity ($u^{*}$) is found by re-arranging the equation using the height of the anemometer. This calculation assumes that the wind speed at 2 m height above the grass field equals that at the same height above the wheat field. The calculated wind speed is then used as input to calculate one-sided *g*_bw_ according to Fig. 2A. Shielded air temperatures were used to correct the diffusion coefficients, according to Appendix 2 in Jones (2013). Weather data are from 2022 in Wageningen (51°58ʹ52.3ʺN, 5°37ʹ13.8ʺE) (https://maq-observations.nl/veenkampen/).

Wind varies significantly from day to day in the field, causing temporal fluctuations of the boundary layer. By combining above-canopy wind profiles (Fig. 7) with the theoretical *g*_b_ relationship (Fig. 2A) and weather-station wind data, *g*_bw_ can be estimated throughout the year (Fig. 8). Values of the characteristic dimension (*L*) of 0.015, 0.0825, and 0.15 m were used, which are typical for a wide range of species, and given that *L* is typically between 0.5 and 0.8 of the maximum leaf dimension in the direction of the wind (Schuepp, 1993), these dimensions are representative of a broad range of agricultural and horticultural species (Cho *et al*., 2007; Evers *et al*., 2007; Moeller *et al*., 2014; Lacube *et al*., 2017; Kimura *et al*., 2020b; Pan *et al*., 2022; Tamang *et al*., 2023).

Leaves with small *L* are more sensitive to wind-speed changes, leading to wider distributions and higher *g*_bw_ compared to leaves with larger *L*, while there is little monthly temporal variation in *g*_bw_ throughout the year. Importantly, these data are 1-minute averages, which smooth out wind gusts; real *g*_bw_ values, especially for small leaves, might fluctuate more and be skewed higher due to turbulence, in which case these values can be multiplied by 1.5 (Jones, 2013). Thus, these distributions only indicate the potential distribution, though they are of similar magnitude as the measured values in the literature (Fig. 1). At the canopy top, values of *g*_bw_ of ∼1.25 mol m^−2^ s^−1^ for thin leaves (*L*=0.015 m) and <0.50 mol m^−2^ s^−1^ for larger leaves (*L*=0.0825 m) appear typical throughout the year. Values of 0.50 mol m^−2^ s^−1^ could limit gas exchange (Fig. 6A), especially lower in the canopy, where wind speed rapidly declines due to the canopy structure (Fig. 7).

### Spatiotemporal dynamics of diffusive limitations of photosynthesis in the field

**Fig. 9.**
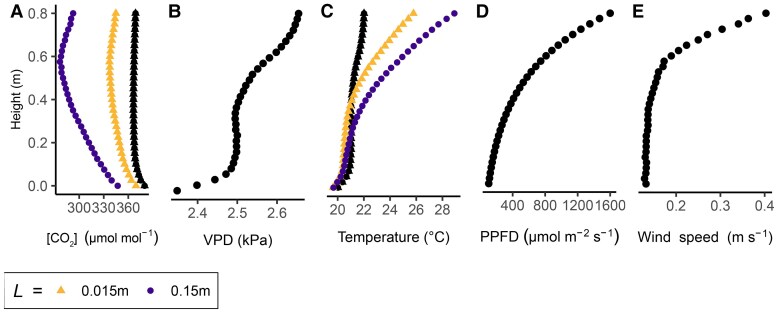
Vertical microclimate profiles of canopy air conditions and leaf surface conditions for leaves that differ in their characteristic leaf dimension. Black symbols are conditions measured within the canopy air of a wheat crop and coloured symbols are the conditions at the leaf surface, calculated according to Aphalo and Jarvis (1993), for two different values of the characteristic leaf dimension, *L*, represented by the mean length (m) in the direction of the wind. The data represented by the black symbols are taken from https://www.tr32db.uni-koeln.de/search/view.php?dataID=1807 and Vilà-Guerau de Arellano *et al*. (2020) using the 15 June 2018 dataset and calculating averages for the time between 12.00 h and 16.00 h. The height of the top of the canopy is 0.8 m. (A) CO_2_ concentration, (B) vapour pressure deficit (VPD), (C) temperature, (D) incident photosynthetically active radiation, shown as the photosynthetic photon flux density (PPFD), and (E) horizontal wind speed. Leaf surface conditions were calculated from model output: the parameters used can be found in Fig. 10.

Here, we consider vertical profiles within the canopy of a wheat crop during a June afternoon with low wind (Fig. 9). This scenario is used as a snapshot to explore the effects of microclimatic variation and the decline of g_bw_ within the canopy. Climatic variables can be altered in the online version of the model at www.plantgasexchange.com. Measured data of the vertical microclimate in wheat were taken from Vilà-Guerau de Arellano *et al*. (2020) and represent the peak of the growth season with high photosynthetic activity. A typical photosynthetic photon flux density (PPFD) profile was calculated based on Burgess *et al*. (2015), using their vertical light distribution pattern but rescaled to a canopy height of 0.8 m, assuming a maximal PPFD of 1600 μmol m^−2^ s^−1^ at the top of the canopy (Fig. 9D). Both studies had similar leaf area indices. As the focus in this review is low-wind scenarios and heat and gas exchange processes, modelling of wind-induced movement and its impact on spatiotemporal light distribution with the canopy (windflecks) was not considered, but it plays another important role in optimizing whole-plant canopy photosynthesis (Burgess *et al*., 2019, 2021; Durand and Robson, 2023), and could potentially create scenarios where a high light intensity occurs simultaneously with low wind inside the canopy.

**Fig. 10.**
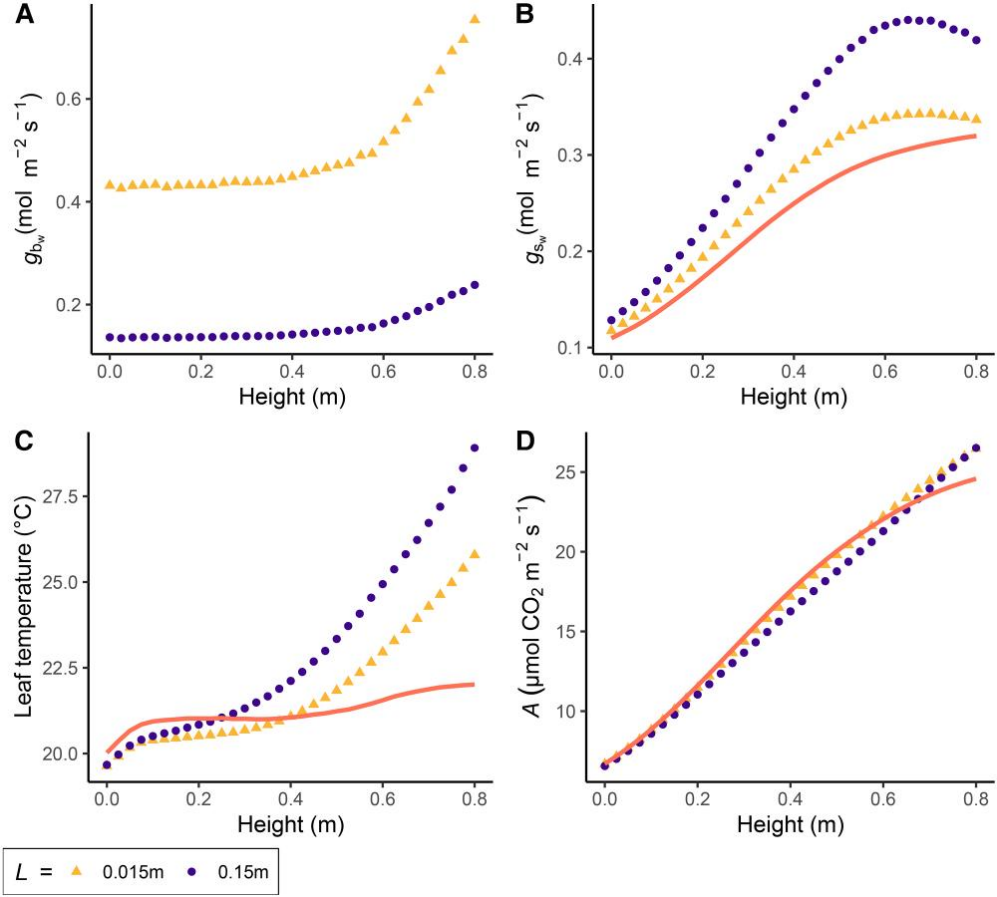
Simulated vertical microclimate profile within a wheat crop derived from a combined model of photosynthesis, stomatal conductance, and transpiration, for leaves that differ in their characteristic leaf dimension. The model integrates key physiological processes to predict gas exchange and energy balance responses under steady-state conditions. The model is equivalent to the one used in Figs 5 and 6 with the exceptions that (1) it is in steady state and solved with a nested iterative procedure (Newton–Raphson method) (Kim and Lieth, 2003) instead of an ordinary differential equations solver, (2) it has a simplified energy balance (https://www.licor.com/support/LI-6800/topics/equation-summary.html), and (3) the Michaelis constants *K*_c_ for CO_2_ and *K*_o_ for O_2_ were actualized for wheat instead of tobacco (Silva-Pérez *et al*., 2017). The characteristic leaf dimension, *L*, is represented by the mean length (m) in the direction of the wind. (A) Single-sided boundary layer conductance for water vapour (*g*_bw_). In (B–D) the orange line represents an infinite *g*_bw_ scenario. (B) Two-sided stomatal conductance for water vapour (*g*_sw_), (C) leaf temperature, and (D) net photosynthesis rate (*A*). Parameters: *V*_cmax25_=140 μmol CO_2_ m^−2^ s^−1^, where ‘25’ indicates standardization to a temperature of 25 °C; *J*_max25_=220 μmol CO_2_ m^−2^ s^−1^ (McAusland *et al*., 2020); *g*_m25_=5 μmol m^−2^ s^−1^ Pa^−1^ (Tazoe *et al*., 2009); *R*_d25_=0.9 μmol CO_2_ m^−2^ s^−1^ (with temperature corrections from Sharkey *et al*., 2007); Abs_PAR_=0.86; Abs_shortwave_=0.5; Curvature *J*/*PAR*=0.7; conversion factor from μmol m^−2^ s^−1^ to W m^−2^, *k*_sun_ calculated as $\frac{1}{\frac{500}{120}\times 0.47}$ (Stanghellini *et al*., 2019); nocturnal stomatal conductance $g_{0}$=0.02; slope of A/$g_{s}$, *g*_1_=7.38; $D_{0}$=2 sensitivity factor for $D_{s}\;$response, *D*_0_=2 (Leuning, 1995).

The vertical gradients of PPFD and wind speed (Fig. 9D, E) are more substantial than those of [CO_2_], [H_2_O], and temperature (Fig. 9A–C). Still, it is noteworthy that the [CO_2_] of the air is 366 μmol mol^−1^ at the top of the canopy and increases non-linearly towards 380 μmol mol^−1^ at the bottom of the canopy, making a range of 14 μmol mol^−1^. This seemingly minor microclimatic vertical gradient and depletion relative to the air above the canopy becomes amplified when modelled at the leaf surface, taking into account *g*_b_ (Fig. 10A), and is more substantial for larger leaves. For example, in the extreme scenario of *L*=0.15 m, [CO_2_] at the leaf surface is 277 μmol mol^−1^ just below the top of the canopy and increases towards 347 μmol mol^−1^ the bottom, a range of 70 μmol mol^−1^. An opposite trend is observed for leaf temperature, with an increase of heat rather than a decrease (Fig. 9C).

We now shift the focus to the modelled outcomes of the response variables. The *g*_bw_ decreases with depth, an effect more pronounced in smaller leaves compared to larger leaves (Fig. 10A). Both *g*_sw_ and leaf temperature are higher in larger leaves (Fig. 10B, C), corresponding to lower *g*_bw_ but the net photosynthetic rate is slightly higher in smaller leaves for most of the profile (Fig. 10D), due to the higher [CO_2_] at the leaf surface (Fig. 9A), but the difference becomes minimal at the top of the canopy due to stomatal closure. An infinite *g*_bw_ scenario, represented by the solid orange lines in Fig. 10, underscores the potential inaccuracies of this assumption, particularly in the upper canopy where PPFD is highest. For example, the range of leaf temperature in the infinite scenario is only 2 °C, but when *g*_b_ is included it is 6 °C and 9 °C for *L*=0.015 m and *L*=0.15 m, respectively. The model used here only highlights the effect of the combination of microclimatic gradients and *g*_b_ and does not represent whole-canopy photosynthesis, as factors such as water status and physiological differences with height (e.g. nitrogen distribution) are not considered, and are outside the scope of this review.

## Future perspectives

### Boundary layer limitation on photosynthesis: an underestimated impact

In greenhouses, increasing airflow can enhance diurnal photosynthesis by up to 23% (Figs 2, 5, 6). However, these are immediate responses and how microclimatic acclimation occurs and whether such changes produce sustained benefits or introduce trade-offs remains largely unknown. Lack of data for boundary-layer and airflow in CEA make it unclear how often severe $g_{b}$ limitations occur, but low wind speeds and obstructive canopies suggest that they might be common. In field crops, low $g_{b}$ commonly occurs when large leaves experience low wind. Under such circumstances, aerodynamic coupling weakens, and minor canopy air gradients become amplified at the leaf surface, affecting photosynthesis and stomatal regulation (Figs 8, 10). But real-leaf complexities remain under-represented in current models, and hence further empirical validation is required as plant traits can mitigate these limitations.

### Dynamic environmental control of the boundary layer in indoor agriculture

In controlled environments, $g_{b}$ is easier to manipulate via airflow than in the field (Kimura *et al*., 2020b; Boekee *et al*., 2023), making it a promising target for real-time climate manipulation. However, this potential is hindered by the following major issues.

(1) Limited experimental data: datasets examining the impact of $g_{b}$ on photosynthesis at low wind speeds under undisturbed microclimatic conditions and over the diurnal cycle are non-existent.

(2) Difficulties in validation: gas exchange data are typically collected under conditions of high $g_{b}$ in chambers where the energy balance is altered (Still *et al*., 2019). This necessitates the use of alternative methods such as measuring chlorophyll fluorescence and by solving energy balance equations in combination with thermal imaging, These methods are suitable for long-term and spatial assessment without disturbing the boundary layer.

(3) Choice of control strategy: there is no consensus on the type or direction of airflow that is most efficient; however, turbulence might be a more effective strategy for air mixing than uniform flow since it enhances transport processes (Jones, 2013).

(4) Cost-effectiveness: forced air circulation increases energy use, and dehumidification is an additional cost in (semi-)closed systems (Stanghellini and Katzin, 2024).

### Understanding long-term acclimation is key to increasing photosynthesis

Short-term $g_{b}$ responses are quantifiable for both field-grown and CEA crops. On the other hand, long-term microclimatic acclimation to altered airflow, via changes in stomatal behaviour, leaf traits, or energy-balance regulation, is poorly understood, and it could counteract positive effects of $g_{b}$ on the CO_2_ supply chain (Grace, 1988; Smith and Ennos, 2003; Anten *et al*., 2010). For example, growth under elevated vapour pressure deficit can lead to reduced $g_{s}$ and $g_{m}$ (Du *et al*., 2019), whilst plants grown under increased airflow can exhibit better tolerance to sudden drops in humidity due to improved stomatal functionality (Carvalho *et al*., 2015). In the field, more variable conditions could produce even more complex interactions that are not yet understood (Vialet-Chabrand *et al*., 2024). Priorities for future experimental work include the following.

(1) Distinguishing between immediate and acclimated responses (Busch *et al*., 2024).

(2) Improving measurement standards. For light intensity, researchers routinely measure PAR at the top of the crop canopy and simple equations can model the extinction profile. For wind, on the other hand, such models only exist for outdoor canopies and not for smaller canopies. They also require more parameters related to the crop architecture that are difficult to generalize. Because no standard methods or simplified models exist, a wide range of measurement approaches are currently used (Box 1).

(3) Focusing on low airflows. Research is needed on low wind speeds (<2 m s^−1^) in order to isolate microclimatic acclimation from the mechanical acclimation that often occurs in the field (Anten *et al*., 2010).

### From simplified physics to transport from real leaves in the field and controlled environments

Models of heat and gas exchange often rely on idealized assumptions such as uniform canopy air, smooth leaf surfaces, and static architectures that overlook the spatial and temporal heterogeneity of the real leaf environment, especially under low wind. While some account for the leaf boundary layer (Tuzet *et al*., 2003; Wu *et al*., 2019), few models integrate the impact of plant architecture on both $g_{b}\;$and aerodynamic conductance ($g_{a}$). In this review, we have isolated leaves from the canopy to focus solely on leaf-level processes and ignored the role of these functional–structural interactions. This could be addressed by integrating computational fluid dynamics with functional–structural plant models to couple the plant responses to the air climate and to guide airflow control based on plant architecture, thereby enabling targeted, crop-specific climate manipulation (Fatnassi *et al*., 2023). At the leaf level, traits remain underexplored and new tools that quantify $g_{b}$ of real leaves should help to clarify their impact, thereby assisting in developing strategies to control the boundary layer, both for field agriculture and CEA.

## Conclusion

The boundary layer remains a hidden bottleneck in our understanding of photosynthesis limitations. It is well-characterized in theory, but rarely integrated into plant physiological research or crop optimization. This review highlights the need to reconceptualize $g_{b}$ not as a passive by-product of airflow but as an active and manipulable constraint in both field and controlled environments. Model predictions and literature data demonstrate that low $g_{b}$ results in a complex interplay with a large impact on leaf microclimate and limitation of photosynthesis of up to 23%, which depends on stomatal sensitivity to the microclimate, photosynthetic capacity, and the temperature response. This review also highlights the need to revisit and update existing models to better quantify the influence of the boundary layer using current data and methodologies. Future research should focus on developing measurement methods and models that integrate the complexity of $g_{b}\;$interactions under realistic conditions, especially at low wind speeds where its influence is most pronounced. Integrating leaf-level complexity, plant architecture, and canopy microclimate into future experimental and modelling approaches will be key to refining what are currently mostly empirical models, and will enable better-informed decisions in crop management and will potentially guide genetic targeting for improving photosynthesis.

## Data Availability

A non-steady state version of the combined photosynthesis, stomatal conductance, and energy balance model is available in Julia using differential equations (ODE solver) and a steady-state version using a nested iterative procedure (Newton–Raphson method) is available in R on GitHub (https://github.com/KillianWUR/Review_paper_wind_photosynthesis). An interactive webpage featuring the model is available at www.plantgasexchange.com, with the diurnal model on the subdomain https://diurnalmodel.plantgasexchange.com and the profile model on https://profilemodel.plantgasexchange.com.
